# Inflammatory and interferon gene expression signatures in patients with mitochondrial disease

**DOI:** 10.1186/s12967-023-04180-w

**Published:** 2023-05-19

**Authors:** Emily B. Warren, Eliza M. Gordon-Lipkin, Foo Cheung, Jinguo Chen, Amrita Mukherjee, Richard Apps, John S. Tsang, Jillian Jetmore, Melissa L. Schlein, Shannon Kruk, Yuanjiu Lei, A. Phillip West, Peter J. McGuire

**Affiliations:** 1 Metabolism, Infection and Immunity Section, National Human Genome Research Institute, National Institutes of Health, Bethesda, MD USA; 2 Center for Human Immunology, National Institutes of Health, Bethesda, MD USA; 3 Department of Immunobiology, School of Medicine, Yale University, New Haven, CT USA; 4 Department of Microbial Pathogenesis & Immunology, Texas A&M University, Bryan, TX USA

**Keywords:** Anti-viral signaling, Inflammation, Interferon, Mitochondrial disease, PBMCs

## Abstract

**Background:**

People with mitochondrial disease (MtD) are susceptible to metabolic decompensation and neurological symptom progression in response to an infection. Increasing evidence suggests that mitochondrial dysfunction may cause chronic inflammation, which may promote hyper-responsiveness to pathogens and neurodegeneration. We sought to examine transcriptional changes between MtD patients and healthy controls to identify common gene signatures of immune dysregulation in MtD.

**Methods:**

We collected whole blood from a cohort of MtD patients and healthy controls and performed RNAseq to examine transcriptomic differences. We performed GSEA analyses to compare our findings against existing studies to identify commonly dysregulated pathways.

**Results:**

Gene sets involved in inflammatory signaling, including type I interferons, interleukin-1β and antiviral responses, are enriched in MtD patients compared to controls. Monocyte and dendritic cell gene clusters are also enriched in MtD patients, while T cell and B cell gene sets are negatively enriched. The enrichment of antiviral response corresponds with an independent set of MELAS patients, and two mouse models of mtDNA dysfunction.

**Conclusions:**

Through the convergence of our results, we demonstrate translational evidence of systemic peripheral inflammation arising from MtD, predominantly through antiviral response gene sets. This provides key evidence linking mitochondrial dysfunction to inflammation, which may contribute to the pathogenesis of primary MtD and other chronic inflammatory disorders associated with mitochondrial dysfunction.

**Supplementary Information:**

The online version contains supplementary material available at 10.1186/s12967-023-04180-w.

## Background

In mitochondrial disease (MtD), a bidirectional relationship between MtD and systemic inflammation emerges, wherein mitochondrial dysfunction may trigger inflammatory cascades, which may then reciprocally contribute to the pathogenesis of MtD. Mouse models have linked primary mitochondrial dysfunction and systemic inflammation. *Polg*^*D257A/D257A*^ mutator mice (hereafter: *Polg* mice), which accumulate mtDNA mutations causing a gradual reduction in mitochondrial respiration [1], display aberrant type I interferon (IFN-I) responses in the innate immune axis leading to immunometabolic dysfunction, accelerated aging, and reduced lifespan [2]. The *Ndufs4*^*−/−*^ mouse, a model of neurodegenerative MtD, is also marked by widespread inflammation [3], including increases in serum levels of inflammatory cytokines (IFN-ɣ and IL-6), inflammatory markers in the skin and liver, and numbers of activated microglia [4]. These responses may be initiated in part through mitochondrial components acting as damage-associated molecular patterns (DAMPs) to activate pattern recognition receptor (PRR) signaling, for example mtDNA activation of the cGAS/STING antiviral response or the NLRP3 inflammasome [5–7], which can trigger the production and release of IFN-I and interleukin-1β (IL-1β). In primary MtD, mitochondrial dysfunction may cause these pathways may be continuously activated, leading to chronic inflammation.

Chronic inflammation contributes to numerous disorders, including cardiovascular and metabolic disease, cancer, and neurodegeneration [8, 9]. Neurodegenerative diseases, such as many forms of MtD, may present a uniquely damaging intersection between inflammation and mitochondrial dysfunction. Pro-inflammatory cytokines, released during systemic inflammation, reach the central nervous system (CNS) via multiple pathways including the blood brain barrier, the choroid plexus, and the vagus nerve [10], and may modulate region-specific immune cell activation in the brain [11, 12], leading to microglial activation, cytotoxicity, and immune dysregulation [10, 13]. Microglial activation releases cytokines, such as TNFɑ, which impair neuronal mitochondria, causing both oxidative stress and activating additional inflammatory signaling [14]. Recent studies have demonstrated that depletion of leukocytes, including microglia, abrogates neuronal death in the *Ndufs4*^−/−^ mouse [15, 16]. Consequently, primary mitochondrial defects may initiate systemic and CNS inflammation, which may contribute to neuronal damage observed in patients with MtD, which manifests clinically as seizures, developmental regression, and degeneration.

To date, most studies on systemic inflammation in MtD have been performed in model organisms. In this study, we aimed to determine if increased inflammation or inflammatory signaling was also observable in human MtD. We performed RNAseq on peripheral blood mononuclear cells (PBMCs) in a heterogeneous group of patients with MtD and controls, using gene set enrichment analysis (GSEA) to identify positively and negatively enriched transcriptional signatures in MtD. GSEA has been extensively validated as a method to identify patterns of gene expression with robust biological relevance [17]. We compared those RNA signatures with those from transcriptomic studies of mitochondrial encephalomyopathy, lactic acidosis, and stroke-like episode (MELAS) patients and two mouse models of mitochondrial dysfunction. Across all four studies we observed enrichment of immune activation and inflammatory gene sets, particularly in antiviral pathways.

## Methods

### Participants

All 81 participants were consented and enrolled in an IRB approved longitudinal natural history study of viral infection and immunity in children with MtD (NIH MINI Study, NCT01780168, www.clinicaltrials.gov) and evaluated at the NIH Clinical Center. Participant characteristics are shown in Table 1. The diagnosis of MtD was made by the referring provider (i.e., neurologist, clinical geneticist), and Modified Walker criteria score of “probable” (P) or “definite” (D) was assigned. The mean age of the control and MtD cohorts were 14.2 (Std dev = 10.6) and 18.4 (Std dev = 16.1) years of age (P = 0.15), respectively. Molecular testing was available for 30 out of 32 patients (94%) with MtD. The MtD cohort was divided into “treated” (n = 22) or “untreated” (n = 9), based on concurrent medications at the time of sampling.  For this study, treatment includes any one or more of the following mitochondria-targeted compounds: Coenzyme Q10 (CoQ10), Sodium bicarbonate, EPI-743, B vitamins (B complex, or B2, B6, B9, or B12), and L-carnitine, collectively termed as “MitoCocktail”. Importantly, MitoCocktail treatment does not exclude the presence of other supplements or medications. “Untreated” indicates the absence of any known MitoCocktail treatments, or other medications or supplements with known direct mitochondrial action.

### Comparative study selection

Transcriptomic datasets were identified and selected from the Gene Expression Omnibus (GEO) (https://www.ncbi.nlm.nih.gov/geo/) database. GEO was queried using search terms for mitochondrial disease, human or mouse organism, and excluding iPSC and cell line studies. Search terms for mitochondrial disease included “mito*”, “mtDNA”, “MELAS”, “MERFF”, “PEO”, “Leigh”, “Alpers”, “Barth”, “CPEO”, “KSS”, “LHON”, “MIRAS”, “MNGIE”, “NARP”, “Polg”, “SANDO”, “SCAD”, and “TK2D”.

### Transcriptomic analysis

PBMC were isolated from 5 mL whole blood using LeucoSep tubes (Greiner Bio-one) and Ficoll-Paque Plus (GE Healthcare) for density gradient centrifugation, before lysis in TRIzol (Thermo Fisher, Waltham, MA). For RNA extraction samples were batched according to their age, gender and case/control status, and two reference samples were simultaneously processed with each batch. Total RNA was isolated and purified with miRNeasy kit (Qiagen, Hilden, Germany), with RNA quality and quantity estimated using Nanodrop (Thermo Scientific, Wilmington, DE) and Agilent 2100 Bioanalyzer (Agilent Technologies, Palo Alto, CA). Stranded cDNA sequencing libraries were generated with TruSeq Stranded mRNA Library Prep Kit (Illumina, San Diego, CA) following the manufacturer’s instructions. Briefly, 500 ng of total RNA was used for mRNA selection. After the reverse transcription to 1st strand cDNA, strand info was reserved with dUTPs during 2nd strand synthesis. The dsDNA fragments then had the addition of a single 'A' base and subsequent ligation of the adapter. The products were then purified and enriched with PCR to create the final cDNA library. The library was qualified with Agilent Bioanalyzer and quantified with Qubit 2.0 fluorometer. The cluster generation and paired-end (2 × 75 bp) sequencing was run on Illumina HiSeq 3000 at NHLBI Sequencing Core. All 81 barcoded samples were pooled for one single run, which yielded at least 25 M passed filter paired reads per sample.

### Computational analysis

Sequence alignment, quality control filtering, and count matrix generation were performed using STAR [18], QoRTs [19], and RSEM [20] on the NIH HPC Biowulf cluster. All subsequent statistical analysis and graphical presentations were performed in R (https://cran.r-project.org/). RSEM-corrected transcript counts were imported using tximport [21], and differentially expressed genes were identified using DESeq2 [22]. Preliminary gene set enrichment analysis (GSEA) was performed using clusterProfiler [23] and visualized using GOplot [24]. Subsequent GSEA analysis used the fgsea [25] package and blood transcription module (BTM) gene sets [26]. Each BTM is a set of genes, which has been shown to show coherent expression across many biological samples [27, 28]. Gene set variation analysis (GSVA) was used to quantify participant level variation in signatures and test correlation with participant age [29] (Additional file 1: Figure S1C). GEO2R and GEOquery [30] were used to perform differential expression analysis from gene expression microarray data in LIMMA [31], whereas additional RNAseq data was analyzed with DESeq2.

## Results

**Fig. 1:**
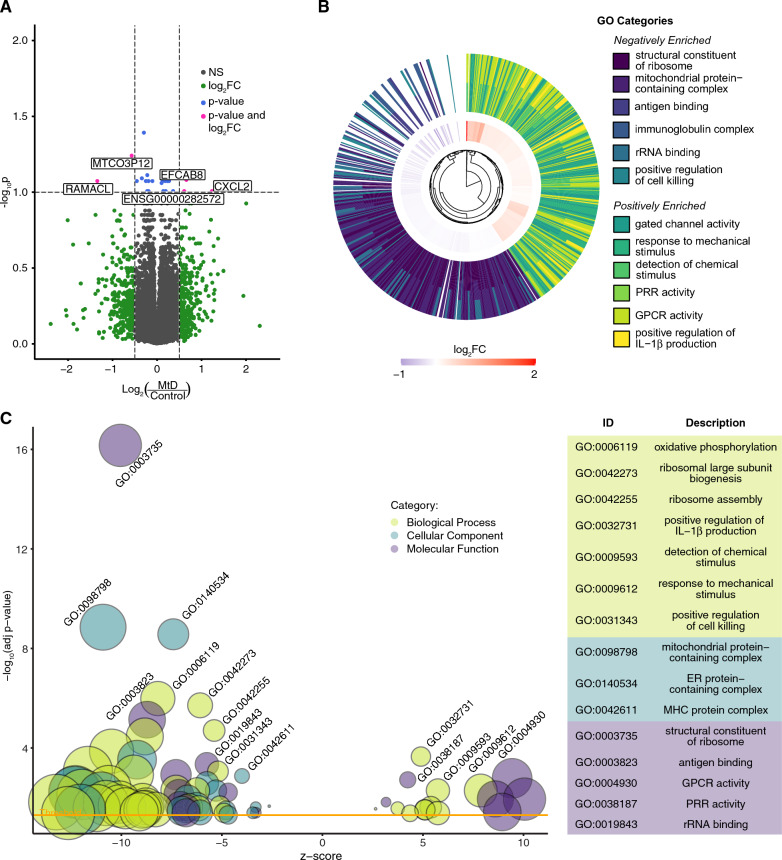
GSEA demonstrates that MtD patients have a positive enrichment in immune activation-associated pathways. **A** Volcano plot of differential expression analysis between MtD (n = 31) and control participants (n = 48). Log_2_FC threshold shown >|0.5|, adjusted p value threshold shown at 0.1. **B** Circular hierarchical cluster plot of top six most significant positive and negative GO categories and associated genes. Inner ring represents log_2_FC of genes belonging to all twelve categories. Outer ring illustrates the GO functional annotation of each gene. **C** Bubble plot of significant GO terms following GSEA analysis and reduction to consolidate redundant terms. Bubble size reflects the size of the gene set, while bubble color reflects the GO category of each set. Z score is derived from the comparison of upregulated and downregulated genes in the set. Yellow line indicates the significance threshold of adjusted p < 0.05. For clarity, only a subset of positive and negative enrichment sets is labeled (see Additional file 3: Table S2 for a full list)

Mouse models of mitochondrial dysfunction have revealed immune signatures marked by elevated inflammation and interferon responses [2, 3, 7, 15]. To understand whether similar transcriptomic signatures occur in people with MtD (Table 1), we performed bulk RNAseq on PBMCs. All participants were in their usual state of health at the time of collection and did not display any symptoms or signs of infection. We examined sample variance using a sample distance correlation matrix of all control participants and MtD patients (Additional file 1: Fig. S1A). Through this analysis, we identified two females (one control, one MtD) that segregated from other samples, and excluded them from subsequent analysis. Using PCA, we observed that our samples do not cluster into diagnosis group (Additional file 1: Fig. S1B). Differential expression analysis identified differentially expressed genes (DEGs) between control and MtD groups (Additional file 2: Table S1). With a threshold of p < 0.1 and log2 fold change (log2FC) of <|0.5|), we detected few DEGs including *CXCL2* (Fig. 1A). By ranking the top 50 DEGs by t statistic, we found that the differences in relative expression between these groups was able to improve clustering of control and MtD groups (Additional file 1: Fig. S1C).

**Table 1:** Patient demographic information

Subject ID	Age at sample	Sex	Age category	Group	Walker score	Gene	MitoCocktail
MINI_01	22.9	F	Adult	Control			
MINI_02	12	M	Ped	Control			
MINI_03	51.49	F	Adult	Control			
MINI_04	7.1	F	Ped	Control			
MINI_05	10.38	F	Ped	Control			
MINI_06	12.82	F	Ped	Control			
MINI_07	3.37	F	Ped	Control			
MINI_08	5.52	M	Ped	Control			
MINI_09	5.62	F	Ped	Control			
MINI_10	8.44	M	Ped	Control			
MINI_11	14.72	M	Ped	Control			
MINI_12	3.99	F	Ped	Control			
MINI_13	6.12	F	Ped	Control			
MINI_14	6.88	F	Ped	Case	D	ATP6	N
MINI_15	14.96	M	Ped	Case	D	PMPCB	Y
MINI_16	14.55	F	Ped	Control			
MINI_17	10.72	F	Ped	Case	D	ATP6	Y
MINI_18	58.38	F	Adult	Case	D	TYMP	Y
MINI_19	53.68	F	Adult	Case	D	mtDNA deletion	N
MINI_20	4.58	M	Ped	Case	D	MT-ND3, MT-ND2	Y
MINI_21	8.71	F	Ped	Case	D	MT-RNR1	Y
MINI_22	20.71	M	Adult	Case	D	NUBPL	Y
MINI_23	35.2	M	Adult	Case	D	ATP6	Y
MINI_24	11.19	M	Ped	Case	D	NDUFS2	Y
MINI_25	6.64	F	Ped	Case	D	HIBCH	Y
MINI_26	34.42	F	Adult	Case	P	COXIII; MT-CYB	Y
MINI_27	10.52	M	Ped	Case	P	MT-CYB	Y
MINI_28	32.82	F	Adult	Control			
MINI_29	35.81	F	Adult	Control			
MINI_30	32.92	M	Adult	Control			
MINI_31	5.87	F	Ped	Control			
MINI_32	9.23	M	Ped	Control			
MINI_33	12.95	M	Ped	Control			
MINI_34	10.88	M	Ped	Control			
MINI_35	4.99	M	Ped	Control			
MINI_36	3.43	M	Ped	Control			
MINI_37	29.18	F	Adult	Case	P	MT-ND4	Y
MINI_38	13.62	F	Ped	Control			
MINI_39	16.27	M	Ped	Control			
MINI_40	15.21	M	Ped	Control			
MINI_41	4.76	F	Ped	Case	D	NUBPL	N
MINI_42	8.38	M	Ped	Case	P	WARS2, RRM2B	Y
MINI_43	11.91	M	Ped	Case	P	MT-RNR2; MT-ND5	Y
MINI_44	2.51	F	Ped	Case	P	unknown	Y
MINI_45	38.39	F	Adult	Case	D	MT-TL1; MT-TS1	Y
MINI_46	7.97	F	Ped	Case	D	MT-TL1; MT-TS1	Y
MINI_47	11.19	M	Ped	Case	P	TK2; COX10; SCO1	N
MINI_48	7.07	M	Ped	Case	D	SDHA	N
MINI_49	14.94	M	Ped	Case	P	MGME1	Y
MINI_50	14.5	F	Ped	Case	P	MT-ND4; MT-TV	Y
MINI_51	6.75	M	Ped	Case	P	NDUFA9; NDUFS2	N
MINI_52	21.85	F	Adult	Case	D	MFN2; MT-ND6	N
MINI_53	47.26	F	Adult	Case	D	MFN2	N
MINI_54	12.04	F	Ped	Case	P	MT-ND1; MT-ND5	Y
MINI_55	51.62	M	Adult	Case	D	MT-ATP6	Y
MINI_56	4.06	M	Ped	Case	D	SURF1	Y
MINI_57	4.82	M	Ped	Case	P	unknown	Y
MINI_58	35.94	F	Adult	Control			
MINI_59	7.05	F	Ped	Control			
MINI_60	3.82	M	Ped	Control			
MINI_61	24.31	F	Adult	Control			
MINI_62	22.61	F	Adult	Control			
MINI_63	15.15	F	Ped	Control			
MINI_64	35.52	M	Adult	Control			
MINI_65	21.97	M	Adult	Control			
MINI_66	21.93	M	Adult	Control			
MINI_67	19.88	M	Adult	Control			
MINI_68	8.5	F	Ped	Control			
MINI_69	3.81	M	Ped	Control			
MINI_70	9.26	M	Ped	Control			
MINI_71	15.45	F	Ped	Control			
MINI_72	10.99	F	Ped	Control			
MINI_73	6.56	F	Ped	Control			
MINI_74	10.79	F	Ped	Control			
MINI_75	4.95	F	Ped	Control			
MINI_76	9.05	M	Ped	Control			
MINI_77	13.86	M	Ped	Case	D	mtDNA deletion	N
MINI_78	8.81	F	Ped	Control			
MINI_79	10.66	F	Ped	Control			
MINI_80	4.95	M	Ped	Control			
MINI_81	8.91	M	Ped	Control

To identify patterns of differential gene expression, we performed GSEA using Gene Ontology (GO) categories. This analysis revealed significant positive and negative enrichment of 172 gene sets (adjusted p < 0.05) (Additional file 3: Table S2). In the six most significant positive and negatively enriched categories, we found decreased expression of genes in ribosomal, mitochondrial, B cell and natural killer (NK cell) categories, and increased expression of genes related to stimulus response, ion channel and G protein-coupled receptor (GPCR) activation, pattern recognition receptors (PRR), and interleukin-1β (IL-1β) production (Fig. 1B). Positively enriched gene sets included many involved in immune activation and signaling, including IL-1 and IL-1β production (n = 6), PRR and Toll-like receptor (TLR) binding (n = 2), IFN-β production (n = 2) and viral response regulation (n = 2). Negatively enriched gene sets included mitochondrial proteins and complexes (n = 28), ribosomes and translation (n = 30), natural killer (NK) cell (n = 9), B cell (n = 3), and major histocompatibility complex (MHC) activity (n = 8) (Fig. 1C).

**Fig. 2:**
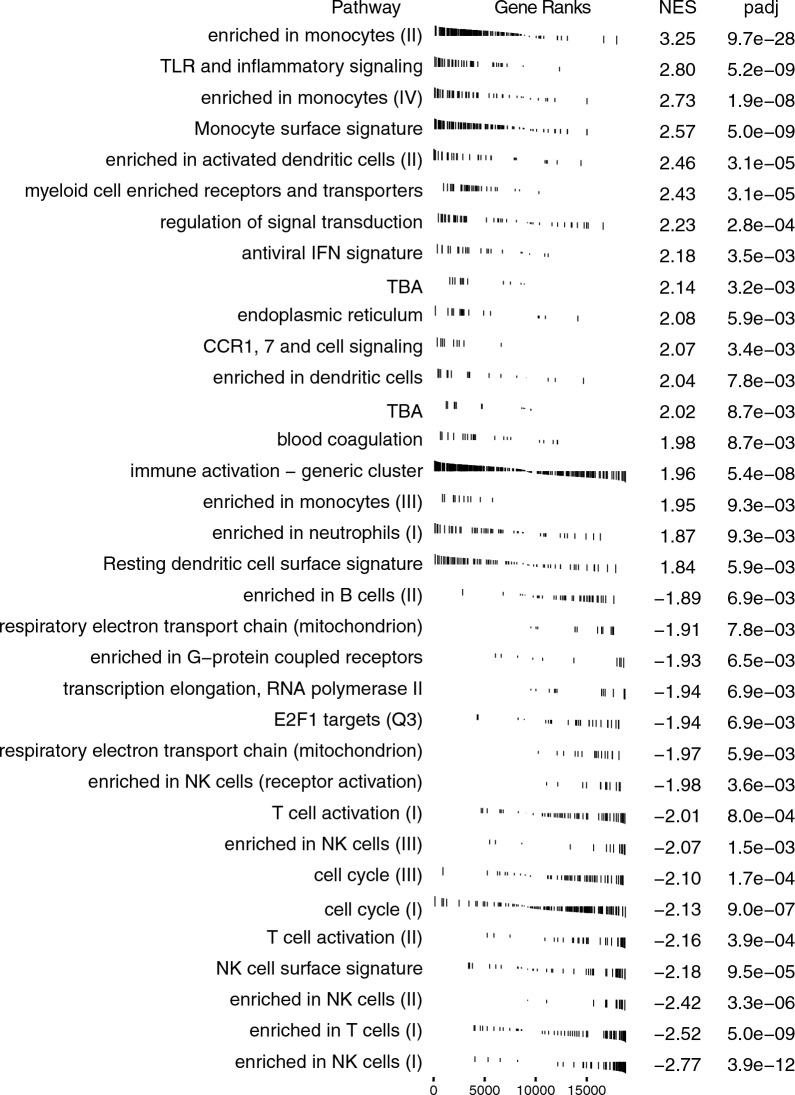
Blood transcriptional module gene ranks. GSEA plots of consolidated BTMs illustrating gene ranks in each transcription set. Left-shifted genes have a positive fold change and are found at the beginning of the gene rank list (positive enrichment), while right-shifted genes have a negative fold change and are found at the end of the gene list (negative enrichment). NES and adjusted p value (padj) for each consolidated BTM indicated at right

As these results suggested immune and translational dysregulation in our MtD patients, we performed GSEA using blood transcriptional modules (BTMs) to identify targeted pathway enrichment [26]. We identified 62 significant gene sets (Additional file 4: Table S3). Examining the consensus pathways from these modules, we observed a positive enrichment in monocyte, TLR, IFN, and immune activation clusters, and negative enrichment in mitochondria, transcription and translation, and NK and T cell clusters (Fig. 2). We also found positive enrichment of inflammatory response, type I IFN response, and innate antiviral response modules, and negative enrichment of plasma cell/immunoglobulins and B cell modules trending toward significance (p ≤ 0.076).

**Fig. 3:**
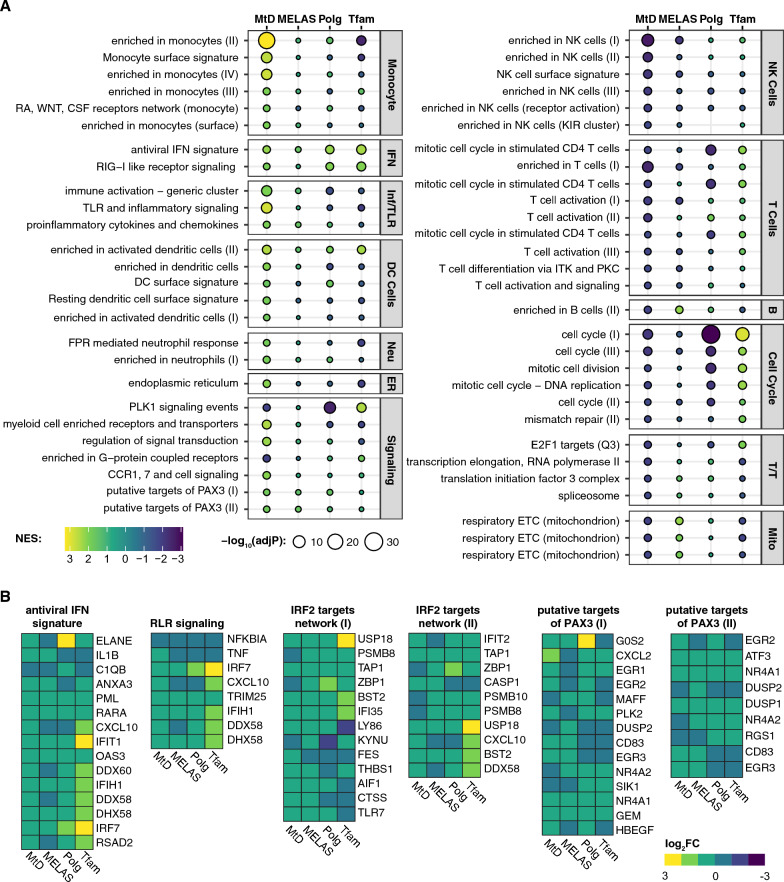
BTM enrichment of MtD patients, MELAS patients, and comparable mouse models of MtD. **A** NES plot of significant BTMs identified in MtD patients. Bubble size represents adjusted p value, bubble color represents normalized enrichment score (NES). Corresponding results from MELAS, *Polg*, and *Tfam* GSEA analysis are shown. Modules are grouped into categories shown at right. IFN: interferon; Inf/TLR: Inflammation and Toll-like receptor signaling; Neu: Neutrophils; B: B cells; T/T: Transcription/Translation; Mito: mitochondrion. See Additional file 4: Table S3 for full results. **B** Log_2_FC of genes from indicated modules

We compared our findings against three other studies: whole blood from MELAS patients [32], bone marrow-derived macrophages (BMDMs) from *Polg* mutator mice [2], and mouse embryonic fibroblasts from *Tfam*^±^ mice [7]. We performed BTM GSEA on these sample sets and compared the enrichment of the previously identified MtD-significant modules (Fig. 3A). Examining mitochondrial clusters confirms previously observed trends—MELAS subjects and *Polg* mice have a positive enrichment, while MtD patients and *Tfam*^±^ MEFs have a negative enrichment. We discovered key overlaps between MtD patients and the previous MELAS study, including negative enrichment in NK cell and T cell modules. Positive enrichment of monocyte, dendritic cells, and neutrophils are unique to MtD patients, though immune and inflammatory cluster enrichment is shared by MtD and MELAS patients. While these modules were not significant in MELAS patients, related viral sensing and IRF2 targets clusters were (Additional file 4: Table S3). Putative PAX3 target gene sets were positively enriched across all four datasets. We examined the genes that compose antiviral and RIG-I like receptor (RLR) (IFN-related), IRF2 targets, and putative PAX3 target modules (Fig. 3B). In IFN gene sets, we observed increased expression of core cluster genes *PML, RARA, IFIT1, OAS3, IFIH1, DHX58, IRF7,* and *TRIM25* in all four datasets*.* In IRF2 targets, which were significantly enriched in MELAS subjects, *Polg* mice, and *Tfam*^±^ MEFs, *USP18, TAP1, BST2, IFI35* were consistently increased. Finally, in putative PAX3 targets, *NR4A1, GEM, ATF3,* and *DUSP1* were increased in all four datasets.

To find explainable sources of variance within our dataset, we considered the sex and age of our participants, as PCA plots suggested that a proportion of variance could be explained by sex (Additional file 1: Fig. S1B). We assessed the contribution of age by performing gene set variation analysis (GSVA) by evaluating a subset of BTMs across our sample population against age (Additional file 1: Fig. S2). We did not observe a significant trend between age and diagnosis group. We divided our samples based on sex and re-performed differential expression analysis. In male samples, we continued to observe poor within-diagnosis sample correlation (Additional file 1: Fig. S3A) and observed that a significant proportion of variance remained unexplained (Additional file 1: Fig. S3B). We observed similar trends in female samples (Additional file 1: Fig. S3C and S3D). In males, we observed that only four DEGs crossed a log2FC >|0.5| and p < 0.1 (Additional file 1: Fig. S4A and Additional file 2: Table S1). Nonetheless, two downregulated genes, *FCRL6* and *KLRC4-KLRK1,* are linked to cytotoxic T and/or NK cells. Ranking DEGs by t-statistic and examining the relative expression of the top 25 genes improved clustering between male control and MtD (Additional file 1: Fig. S4B). In females, no genes were significantly differentially expressed (Additional file 1: Fig. S4C and Additional file 2: Table S1), but we observed within-diagnosis group clustering when examining the t-statistic ranked top 25 DEGs (Additional file 1: Fig. S4D). Ranking by t-statistic and splitting genes into “upregulated” and “downregulated” categories, we examined potential intersections of control and MtD differential gene expression in the full dataset, males, and females. Using the top 500 genes in each category, both upregulated (Additional file 1: Fig. S5A) and downregulated (Additional file 1: Fig. S5B) intersections demonstrated a majority of uniquely identified genes in the full, male, and female datasets, but with larger intersections between the full and male datasets, and the full and female datasets. At the intersection of all three sets, we found 26 upregulated genes and 27 downregulated genes. The upregulated genes include *TMEM106A, TRIM14,* and *LPAR1*, which are associated with positive regulation of NFκB signaling. In contrast, the downregulated genes include *RPL4P4, RPS7P1, RPL13AP5, RPS15P4, RPL7P9*, and *RPL7AP30,* which are processed ribosomal pseudogenes. In aggregate, the function of these genes is unclear, however, as new functions have recently been described for several pseudogenes [33], it is possible that these genes are related to dysregulated ribosomal or RNA processing patterns observed in the GSEA (Additional file 3: Table S2).

**Fig. 4:**
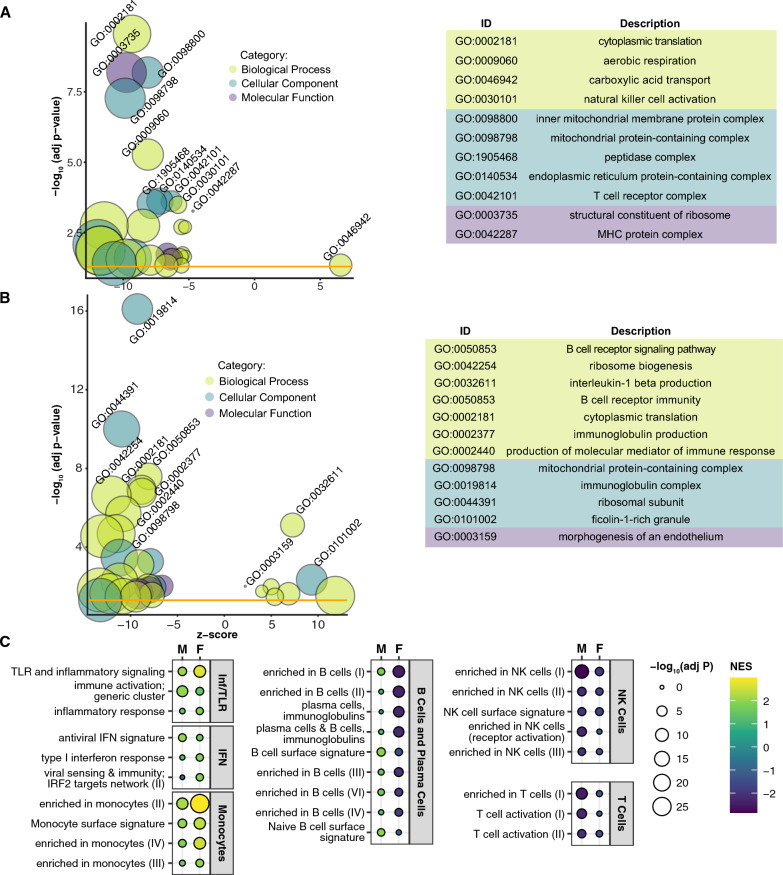
Male and female MtD patients show differential enrichment of immune and inflammatory gene sets. **A** Bubble plot of male controls (n = 22) and MtD patients (n = 16), **B** of female controls (n = 27) and MtD patients (n = 16) shows significant GO terms following GSEA analysis and consolidation of redundant terms. **A**, **B** Bubble size reflects the size of the gene set, while bubble color reflects the GO category of each set. Z score is derived from the comparison of upregulated and downregulated genes in the set. Yellow line indicates the significance threshold of adjusted p < 0.05. See Additional file 3: Table S2 for a full list of significant GO terms. **C** NES plots from selected modules from males (M) and females (F). Bubble size reflects adjusted p value, bubble color reflects NES. Modules are grouped into categories shown at right. IFN; interferon, Inf/TLR; Inflammation and Toll-like receptor signaling. See Additional file 4: Table S3 for full results from all datasets

In males, GSEA of GO terms identified 87 significant gene sets, including negative enrichment of gene sets relating to ribosomes (n = 12), mitochondria (n = 23), MHCs (n = 2), NK cells (n = 9) and T cells (n = 3) (Fig. 4A and Additional file 3: Table S2). In females, significant GO GSEA results (n = 78) revealed similar negatively enriched gene sets, including ribosomes (n = 21), mitochondria (n = 3), and B cells (n = 5), but also positively enriched gene sets including IL-1β production (n = 6) and endothelial morphogenesis (n = 2) (Fig. 4B and Additional file 3: Table S2). Based on these disparate results, we compared male and female GSEA using BTMs. While there were many overlapping significantly enriched categories (Additional file 4: Table S3), we observed intriguing differences between the two groups (Fig. 4C). TLR and inflammatory signaling and monocyte gene sets are more positively enriched in female MtD samples than males, and IRF2 targets are significantly enriched in females. In contrast, several B cell gene sets are significantly negatively enriched in females, whereas these sets are either nonsignificant or positively enriched in males. Finally, we observed that while NK and T cell gene sets are negatively enriched in males and females, these sets were more significant in males.

**Fig. 5:**
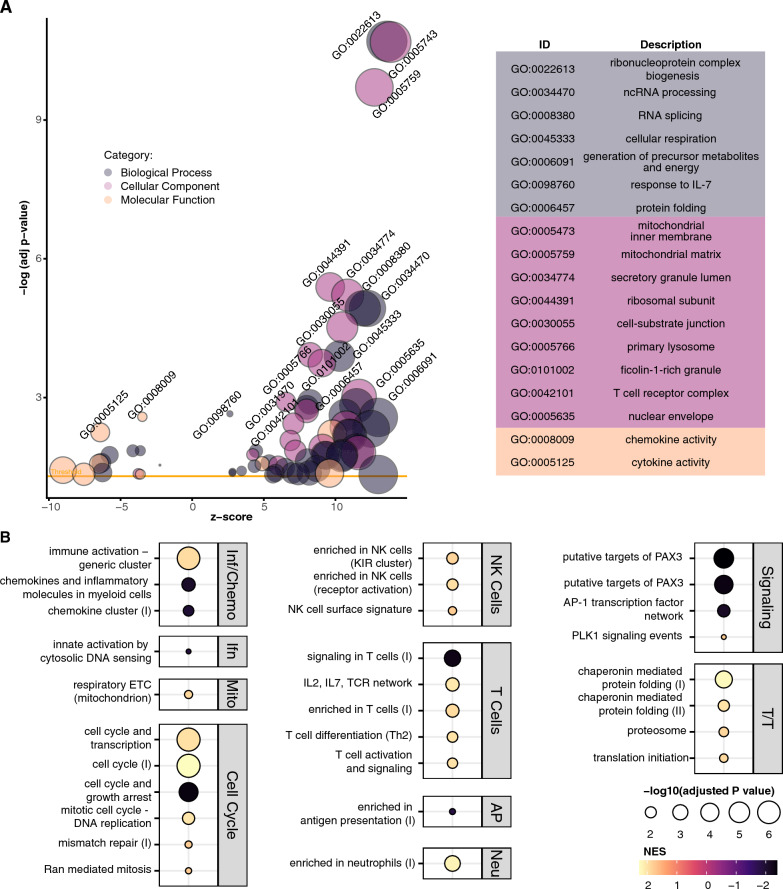
Positive enrichment of mitochondrial, NK cell, and T cell gene sets in MtD patients treated with MitoCocktail. GSEA analysis of MtD patients treated with MitoCocktail (n = 22) against MtD patients not undergoing treatment (n = 9). **A** Bubble plot of significant GO terms in treated MtD patients following GSEA analysis and reduction to consolidate redundant terms. Bubble size reflects the size of the gene set, while bubble color reflects the GO category of each set. Z score is derived from the comparison of upregulated and downregulated genes in the set. Yellow line indicates the significance threshold of adjusted p < 0.05. For clarity, only a subset of positive and negative enrichment sets is labeled (see Additional file 3: Table S2 for a full list). **B** NES plot of a subset of significant BTMs identified in treated MtD patients. Bubble size represents adjusted p value, bubble color represents normalized enrichment score (NES). Modules are grouped into categories shown at right. Inf/Chemo: Inflammation and Chemokines; Ifn: Interferon/Antiviral Sensing; Neu: Neutrophils; Mito: mitochondrion; T/T: Transcription/Translation. See Additional file 4: Table S3 for full results

We further grouped MtD patients into “treated” (n = 22) and “untreated” (n = 9) conditions, on the basis of receiving any class of mitochondria-related treatment, or "MitoCocktail" (see Methods for treatment details). While differential gene expression analysis again did not detect any single significant genes (Additional file 2: Table S1), GSEA of GO terms detected significant enrichment of 151 categories (Fig. 5A and Additional file 3: Table S2). MtD patients treated with MitoCocktail had significant positive enrichment of mitochondrial gene sets, including oxidative phosphorylation, mitochondrial translation, and purine metabolism (n = 43), ribonucleoproteins and rRNA (n = 16), RNA and RNA splicing (n = 12), cell division (n = 8), and protein folding and localization (n = 14) related gene sets. We also observed positive enrichment of defense response and viral process (n = 4), T cells (n = 3) and response to IL-7 (n = 2) gene sets. Negatively enriched categories included chemokine and cytokine (n = 3), receptor activity (n = 6) and response to IL-1, negative regulation of NFκB, and MHC protein (n = 1, respectively) gene sets. Pursuing these findings in GSEA of BTM terms (Fig. 5B and Additional file 4: Table S3), we found that treated patients had a majority positive enrichment of NK and T cell gene sets, and positive enrichment of protein folding genes. In contrast, chemokine, cytosolic DNA sensing, and PAX3 target gene sets were negatively enriched.

## Discussion

Our study is among the first to examine the transcriptomic profile of PBMCs from a cohort of MtD patients. We used GSEA to identify key pathway perturbations, and, using both GO terms and validated BTMs, we observed consistent set enrichments. We observed positive enrichment of IL-1β, IFN, and TLR/PRR GO terms, and monocyte, TLR, and IFN signaling BTMs, suggesting elevated basal levels of inflammation in MtD patients. Conversely, we observed negative enrichment of ribosomal proteins, cell killing, and mitochondrial GO terms, and T cell, NK cell, B cell, and transcription and translation BTMs, suggesting suppression of immune system activity coupled with deficits in translation. These data are a valuable resource to inform future study of the mechanisms of peripheral inflammation in patients with MtD.

While we observed few significant DEGs, our sample population is highly heterogenous. We included male and female, adult and pediatric patients and controls, and patients with multiple MtD disease-causing variants, including both mtDNA and nDNA mutations. Few significant DEGs were detected in the MELAS study, despite restricting to adult patients with the same m.A3243G variant [32]. Further, this distinction in study populations likely underlies the oppositional enrichment between MtD and MELAS groups in mitochondrial gene sets. The MELAS study found a positive enrichment of mitochondrial genes, and our confirmation of this finding with our BTM GSEA approach is an important validation of our technique. The significant negative enrichment of mitochondrial genes among our MtD patients may reflect the differing genetic origins of MtD.

Across all four datasets, we observed a positive enrichment of antiviral IFN or viral sensing signaling modules, supporting previous findings. In *Tfam*^±^ MEFs, a pathway was identified by which mitochondrial stress promotes the release of mtDNA into the cytosol, activating the cGAS-STING-IRF3 antiviral response [7]. In *Polg* BMDMs, type I IFN signaling genes are basally elevated [2]. Together, these demonstrated increased IFN signaling and inflammatory signaling in multiple mouse models of mitochondrial dysfunction. We found significant enrichment of similar pathways in our MtD patients and in MELAS, suggesting basal IFN and antiviral pathway hyperactivation may be common mechanisms in MtD. We identified significant enrichment of an “innate activation by cytosolic DNA sensing” module in *Polg* and *Tfam* datasets, but this module was not enriched in either MtD or MELAS. Consequently, while we confirm enrichment of IFN and inflammatory pathways in human MtD, we emphasize that additional research will be needed to characterize underlying mechanisms.

Our dataset allowed us to perform a preliminary analysis of male and female patients with MtD. While sex did not drive all variance observed, we found interesting differences between males and females. In males, T cell and NK cell GO terms were significantly negatively enriched. In females, B cell and immunoglobulin GO terms were negatively enriched, while IL-1β and ficolin-1 gene sets were positively enriched. BTM terms furthered these findings, demonstrating oppositional enrichment of B cell gene sets between males and females, stronger negative enrichment of T cell and NK cell sets in males, and stronger positive enrichment of monocyte and TLR sets in females. This intriguing segregation of enriched pathways suggests potential sex-specific manifestations of immune dysfunction in MtD. Further, we compared MtD patients treated with MitoCocktail against untreated MtD patients. This comparison suggested that patients receiving treatment have positive enrichment of mitochondrial, ribosomal, and RNA processing gene sets, and of NK and T cell gene sets. In contrast, we observed negative enrichment of chemokine, cytosolic DNA sensing, and PAX3 target gene sets. These results are also preliminary, particularly as our treated and untreated groups are unbalanced. However, these enrichments further support the link between mitochondrial dysfunction and immune dysregulation, as they suggest that treatments supporting mitochondrial function affect immune related gene set expression.

Importantly, there are key limitations to this study. Our findings are based on GO and GSEA methods, which are valuable approaches to identifying gene networks and patterns of subtle changes in gene expression in heterogeneous sample populations. However, these findings are limited to gene expression in uninfected MtD patients and controls, and it will nonetheless be essential for future studies to validate these findings with other methods. For example, gene expression changes or perturbations in cytokine levels in the presence or absence of infection may yield more information about a potential mitochondrial dysfunction-induced hyperinflammatory response, as previously suggested by animal models. Further, though our study suggests potential cell-type specific changes in gene expression in MtD, single cell analysis of PBMC cell types or other tissue-specific immune cells may provide more insight into the mechanisms underlying these observations. We have also presented preliminary data suggesting that there are differences in gene expression patterns in male and female MtD, as well as in treated and untreated MtD. We would like to stress that these findings especially should be validated with other methodologies, in larger patient cohorts, and in other models of MtD. Finally, clinical observations have suggested the coincidence of infection and exacerbation of MtD symptoms. Further animal model and clinical studies directed at this relationship may be necessary to further understand MtD progression, and to characterize the role of immune dysfunction.

## Conclusions

Using multiple GSEA approaches, we demonstrate that MtD patients have positively enriched IFN, inflammatory signaling, and monocyte gene sets, and negatively enriched NK cell, T cell, and ribosomal gene sets. Our approach confirms the enrichment of type I IFN signaling in two mouse models of mitochondrial dysfunction and detects a previously unreported enrichment of viral sensing genes in a study of MELAS patients. MtD and mitochondrial dysfunction may induce basal peripheral inflammation, which likely contributes to the susceptibility to elevated inflammatory responses and sepsis in people with MtD. Further, peripheral inflammation exacerbates neuroinflammation and neurodegeneration in animal models [34, 35]. This may point to an important mechanism underlying the acceleration of neurodegeneration following infection in MtD, and the exacerbation of neurodegeneration in disorders with mitochondrial dysfunction.

## Supplementary Information

**Additional file 1: Figure S1.** Sample variance and differential gene expression analysis. **Figure S2.** Gene set-based comparison of age and diagnosis group. **Figure S3.** Male and female sample variance analysis. **Figure S4.** Male and female differential gene expression analysis. **Figure S5.** Intersection of differentially expressed genes across all control-MtD comparisons.

**Additional file 2: Table S1.** Differential gene expression results from DESeq2.

**Additional file 3: Table S2.** GSEA results using GO terms.

**Additional file 4: Table S3.** GSEA results using BTM terms.

## Data Availability

All summary level data supporting the findings of this study are available within the paper and its Supplementary Information. These data have also been deposited into the database of Genotypes and Phenotypes (dbGaP) under accession number phs003299.v1. Individual level data are not openly available due to patient privacy and are available from the corresponding authors under reasonable request and institutional approval. This study also used publicly available datasets from accession numbers GSE171960, GSE14882, and GSE63767 (GEO). Code for the analyses in this study are available at https://github.com/foocheung/mini_study_2019.

## Abbreviations

MtD: Mitochondrial disease
MELAS: Mitochondrial encephalomyopathy, lactic acid, and stroke-like episodes syndrome
IFN: Interferon
DAMP: Damage-associated molecular pattern
PRR: Pattern-recognition receptor
CNS: Central nervous system
PBMCs: Peripheral blood mononuclear cells
GSEA: Gene set enrichment analysis
GSVA: Gene set variant analysis
NES: Normalized enrichment score
NK cell: Natural killer cell
TLR: Toll-like receptor
BTM: Blood transcriptional module
BMDM: Bone marrow-derived macrophage
RLR: RIG-I-like receptor
