# Supplementary material for: Inflammatory and interferon gene expression signatures in patients with mitochondrial disease
Source: J Transl Med. 2023 May 19;21:331. doi: 10.1186/s12967-023-04180-w (PMC10199642; doi:10.1186/s12967-023-04180-w)

Figure S1. Sample variance and differential gene expression analysis. (A) Sample distance correlation matrix of all control and MtD participants. Scale indicates high sample distances in red, and low sample distances in blue. (B) PCA plots of the first two components of control and MtD samples, using the first 500 genes for analysis. Top, sex and diagnosis group are shown. Bottom, age and diagnosis group are shown. (C) Heatmap of variance stabilized transform expression values of top 50 differentially expressed genes (ranked by t statistic). Genes are clustered by diagnosis and similarity of expression values. Gene names indicated at right with HGNC symbols; ENSEMBL gene ID substituted in absence of available symbol.

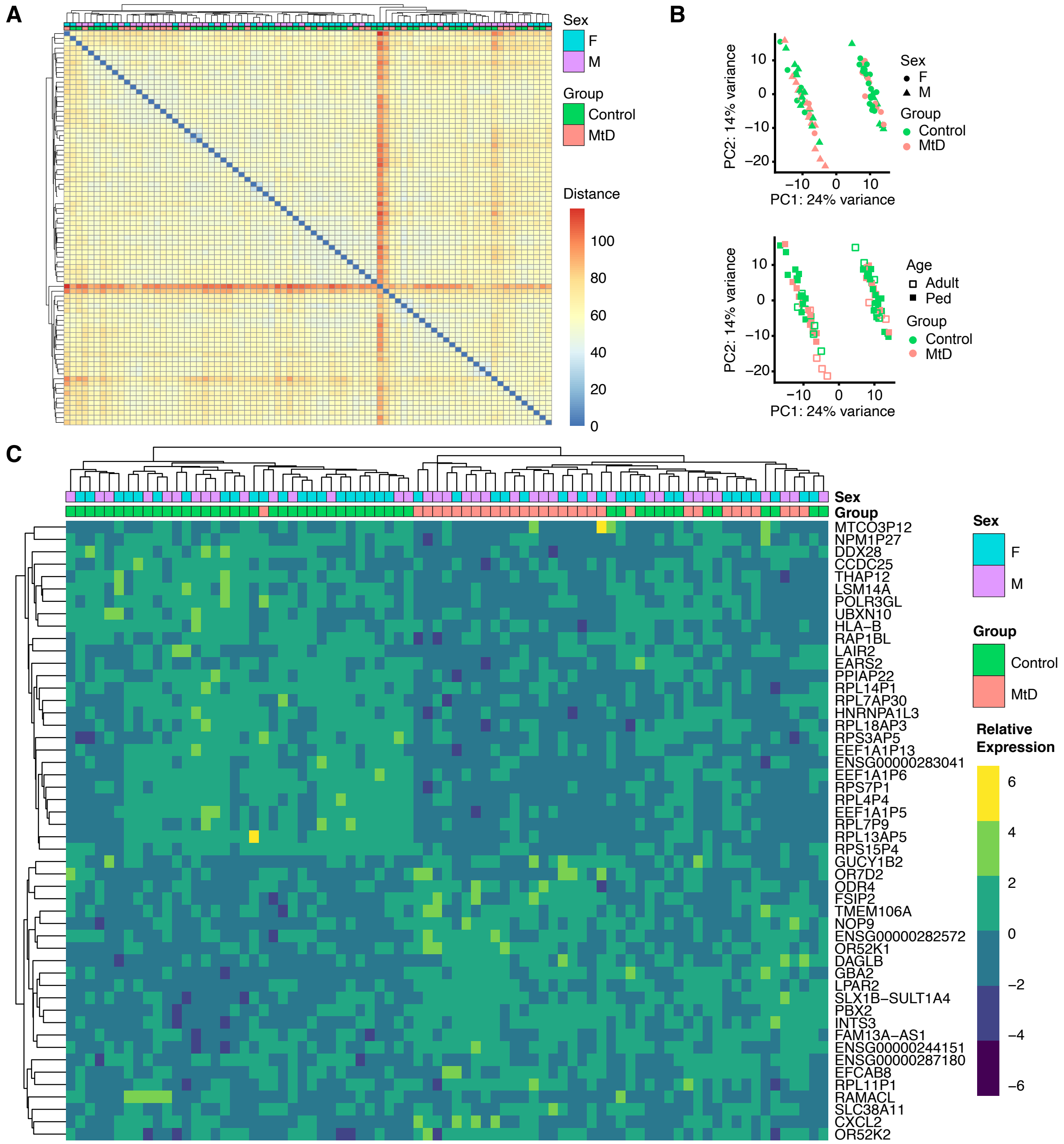

Figure S2. Gene set-based comparison of age and diagnosis group. Gene set variance analysis (GSVA) of a subset of BTM modules, plotting NES for each participant against participant age.

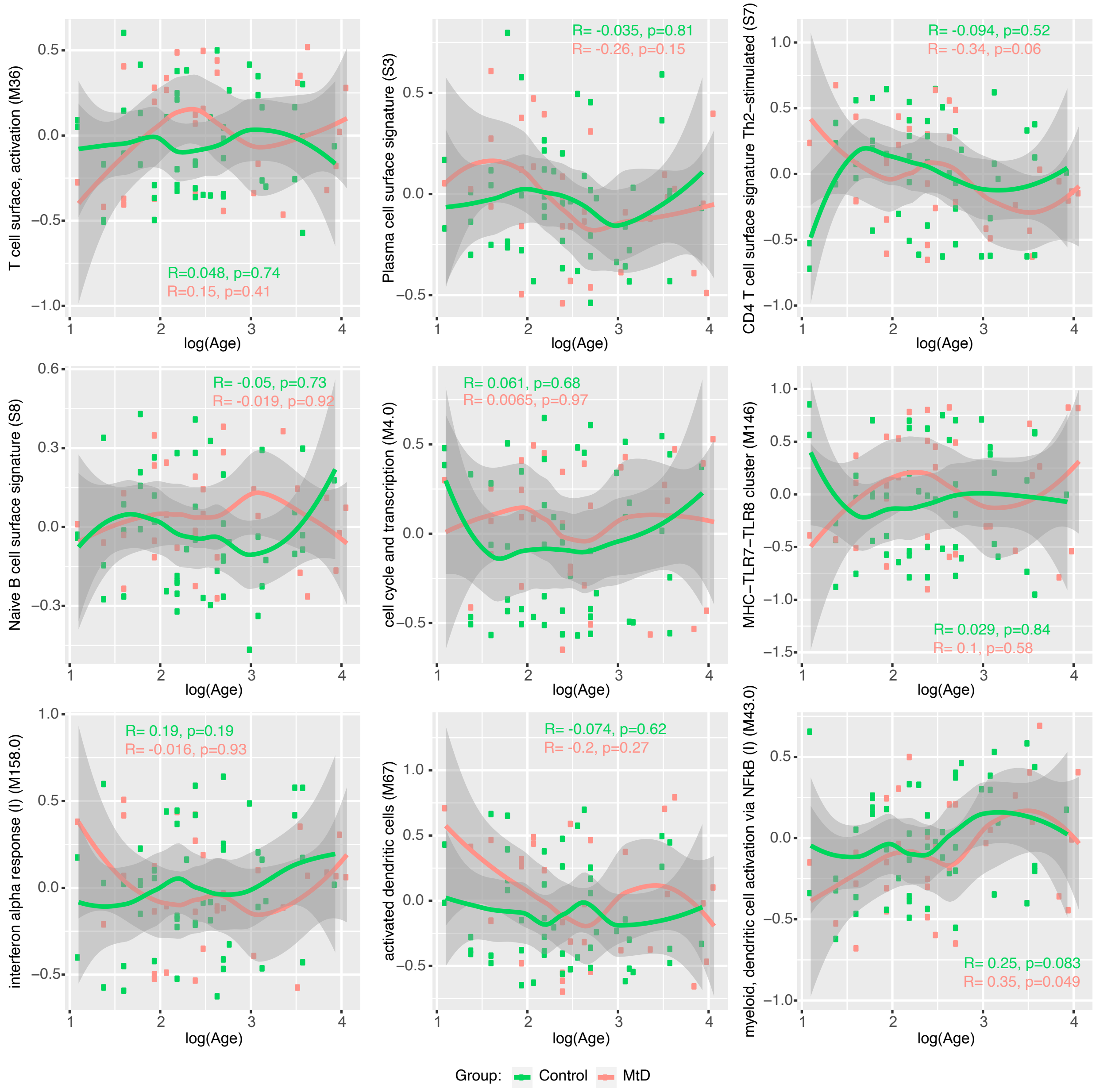

Figure S3. Male and female sample variance analysis. (A) Sample distance correlation matrix of all male control participants (n = 22) and MtD (n = 16) patients. Scale indicates high sample distances in red, and low sample distances in blue. (B) PCA plots of the first two components of male control and MtD samples, using the first 500 genes for analysis. (C) Sample distance correlation matrix of all female control participants (n = 27) and MtD (n = 16) patients. (D) PCA plots of the first two components of female control and MtD samples, using the first 500 genes for analysis.

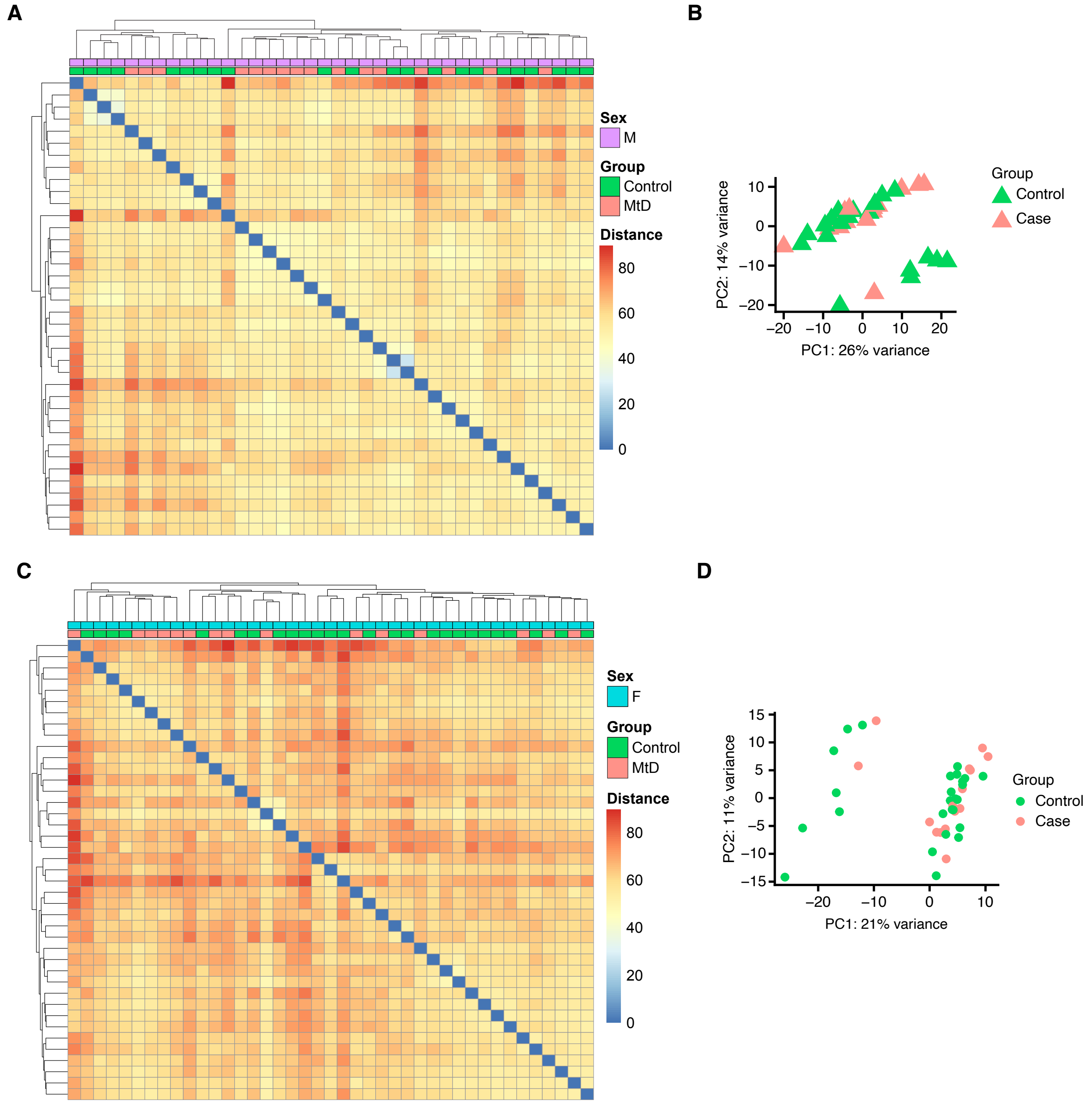

Figure S4. Male and female differential gene expression analysis. (A) Volcano plot demonstrating results of differential gene expression analysis between all male control participants (n = 22) and MtD (n = 16) patients. Significance thresholds depicted at log2 fold change > |0.5|, p value threshold shown at 0.1. (B) Clustered heatmap of top 25 genes ranked by t statistic. Gene names indicated at right with HGNC symbols; ENSEMBL gene ID substituted in absence of available symbol. (C) Volcano plot demonstrating results of differential gene expression analysis between all female control participants (n = 27) and MtD (n = 16) patients. Significance thresholds depicted at log2 fold change > |0.5|, p value threshold shown at 0.1. (B) Clustered heatmap of top 25 genes ranked by t statistic. Gene names indicated at right with HGNC symbols; ENSEMBL gene ID substituted in absence of available symbol.

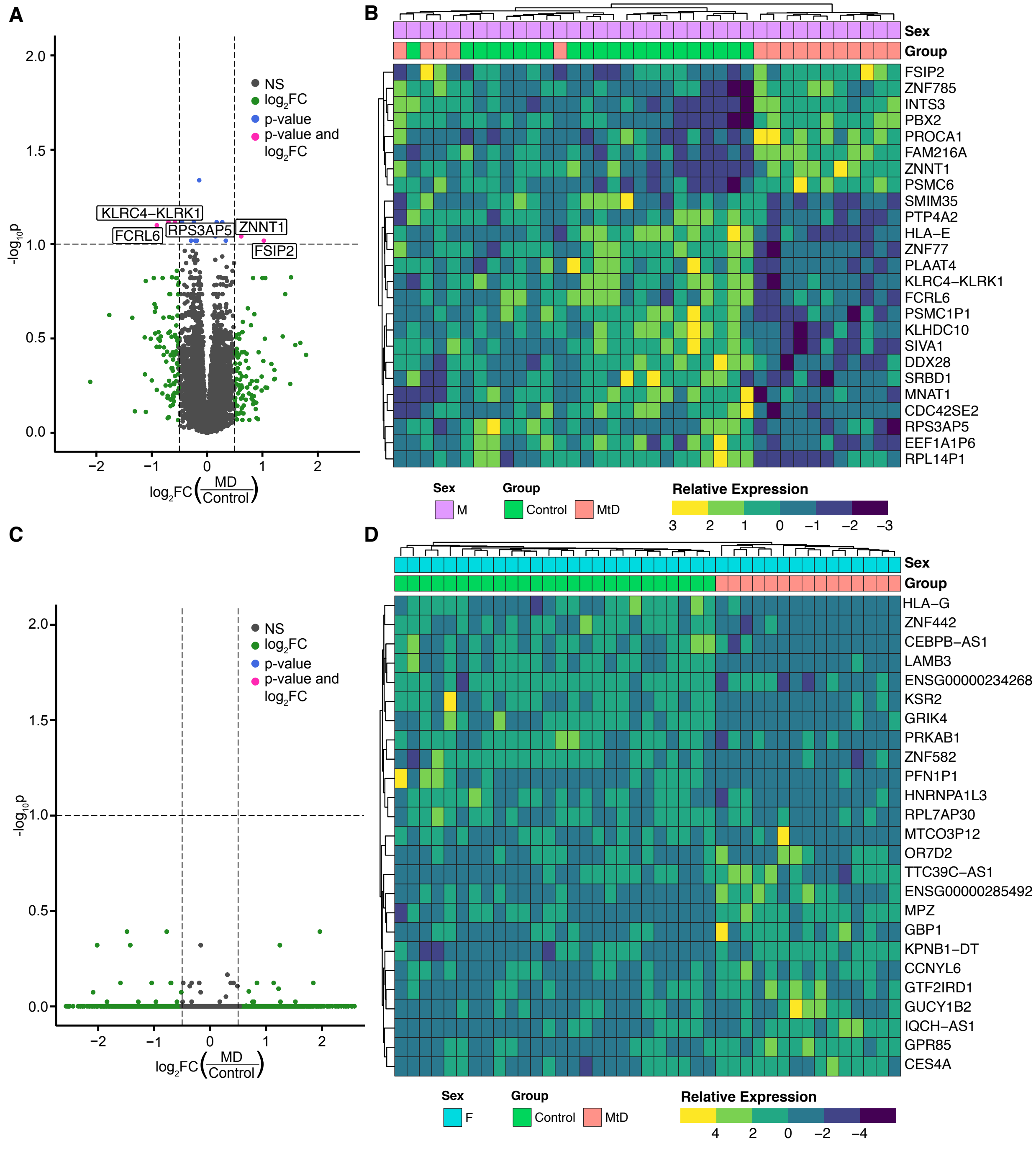

Figure S5. Intersection of differentially expressed genes across all control-MtD comparisons. From all control versus MtD, male control versus male MtD, and female control versus female MtD comparisons, differentially expressed genes were ranked by t statistic and the top 500 genes were chosen. (A) Intersection of upregulated genes between the three comparisons. (B) Intersection of downregulated genes between the three comparisons.

A **B**

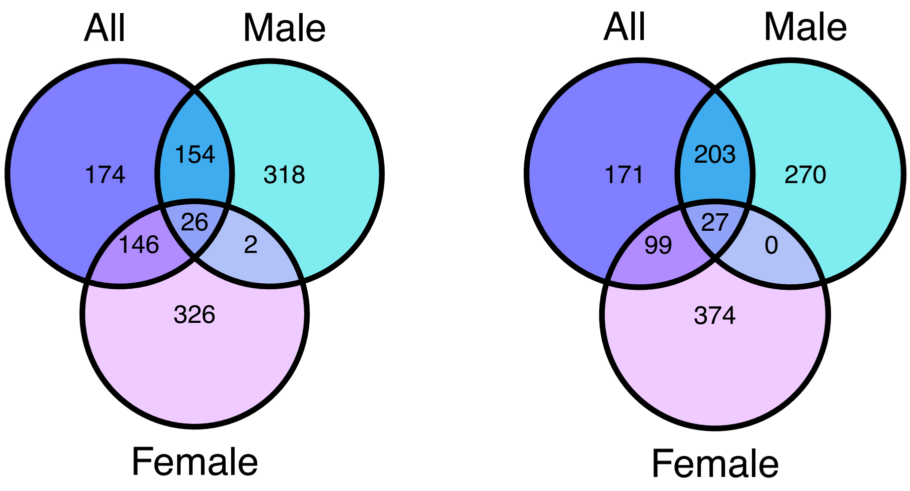

Supplement: Supplementary file 1 — Additional file 1: Figure S1. Sample variance and differential gene expression analysis. Figure S2. Gene set-based comparison of age and diagnosis group. Figure S3. Male and female sample variance analysis. Figure S4. Male and female differential gene expression analysis. Figure S5. Intersection of differentially expressed genes across all control-MtD comparisons. [file 12967_2023_4180_MOESM1_ESM.pdf]
